# Benign Yellow Dot Maculopathy: A Case Series of Patients With a Recently Discovered Macular Phenotype

**DOI:** 10.7759/cureus.74652

**Published:** 2024-11-28

**Authors:** Miguel Santos, Nuno Oliveira, Margarida Baptista, Pedro Arede, João Pedro Marques, Sara Vaz-Pereira

**Affiliations:** 1 Department of Ophthalmology, Unidade Local de Saúde (ULS) Santa Maria, Lisbon, PRT; 2 Department of Ophthalmology, Faculdade de Medicina da Universidade de Lisboa, Lisbon, PRT; 3 Department of Ophthalmology, Unidade Local de Saúde (ULS) Lisboa Ocidental, Lisbon, PRT; 4 Department of Ophthalmology, Unidade Local de Saúde (ULS) Coimbra, Coimbra, PRT

**Keywords:** benign yellow dot maculopathy, inherited retinal diseases, macular dystrophy, multimodal imaging, retina, yellow dot

## Abstract

Benign yellow dot maculopathy (BYDM) is a recently described rare, asymptomatic, early onset, and non-progressive macular phenotype. It is characterized by the presence of multiple white-yellow dots encircling the fovea, which are hyperautofluorescent on fundus autofluorescence. Here, we expand on the few reports available by presenting a case series of five Portuguese patients with clinical BYDM phenotype and congruent multimodal imaging, including the second reported unilateral case. All five patients were female, with a mean age of 31 ± 16 years and mean visual acuity of 0.04 logMAR (logarithm of the minimum angle of resolution), which remained stable throughout long-term follow-up.

## Introduction

In 2017, Dev Borman et al. first described a new macular phenotype: benign yellow dot maculopathy (BYDM) [1]. It is characterized by bilateral or unilateral, numerous small white-yellow dots encircling the fovea [1-3]. BYDM has a childhood onset and is asymptomatic and non-progressive, usually being identified through routine examination [4]. In the six reports available [1-6], optical coherence tomography (OCT) is generally normal, although ellipsoid zone (EZ) and retinal pigment epithelium (RPE) irregularities have been reported [1,5]. All cases display hyperautofluorescence of the dots on fundus autofluorescence (FAF) and normal electroretinography [1,5]. BYDM may be sporadic or follow a presumed autosomal dominant (AD) inheritance (a genetic locus has not yet been identified) [1,3,4].

A range of macular diseases, with variable prognoses, are characterized by white-yellow dots. Differential diagnoses of BYDM include but are not limited to Stargardt disease, Best disease, age-related macular degeneration, autosomal dominant drusen, congenital grouped albinotic spots, drug-induced retinopathies, Bietti crystalline dystrophy, North Carolina macular dystrophy, and oxalosis. It is therefore essential to expand on the body of knowledge of the 46 BYDM cases published thus far [5]. We aim to characterize a group of Portuguese patients with features of BYDM.

## Case presentation

Five patients with clinical features of BYDM were included, originating from three Portuguese centers. Informed consent was obtained. All patients underwent multimodal imaging with color fundus photography, OCT, and FAF, and all, except patient #3, underwent OCT angiography (OCT-A). Demographic and clinical characterization of the cohort is presented in Table 1 and Table 2, respectively.

**Table 1:** Demographic characterization of the BYDM cohort. BYDM: benign yellow dot maculopathy; SD: standard deviation; VA: visual acuity; logMAR: logarithm of the minimum angle of resolution.

Demographic feature	
Number of patients (number of families)	5 (4)
Female gender, n (%)	5 (100)
Age, mean ± SD (years)	31 ± 16.26
Follow-up, mean ± SD (years)	5.8 ± 3.96
VA, mean ± SD (years) (LogMAR)	0.04 ± 0.09

**Table 2:** Clinical features of the included patients. BYDM: benign yellow dot maculopathy; n: number of family; VA: visual acuity; logMAR: logarithm of the minimum angle of resolution.

Clinical features	Patients				
	#1	#2	#3	#4	#5
Family (n)	1	1	2	3	4
Gender	Female	Female	Female	Female	Female
Age (years)	59	24	19	31	22
Follow-up (years)	8	9	2	1	9
Mean baseline VA (logMAR)	0.0	0.0	0.2	0.0	0.0
Mean last visit VA (logMAR)	0.0	0.0	0.2	0.0	0.0
Ocular history	Fuchs endothelial dystrophy	Hypomelanosis of Ito	Myopia	–	Myopia
BYDM laterality	Bilateral	Bilateral	Bilateral	Bilateral	Unilateral

Patients #1 and #2 were, respectively, mother and daughter, with no known history of consanguinity. Visual acuity (VA) was normal in four patients, while patient #3 had slightly reduced VA bilaterally, unrelated to the macular findings. VA remained unchanged during follow-up in all patients. The yellow dots were mostly incidental findings, the exception being patient #2 who was studied after the findings of patient #1. Fundoscopy revealed multiple small yellow dots at the level of the RPE around the fovea. These findings were bilateral in four of the five patients, except for patient #5 who had findings in the right eye only.

**Figure 1:**
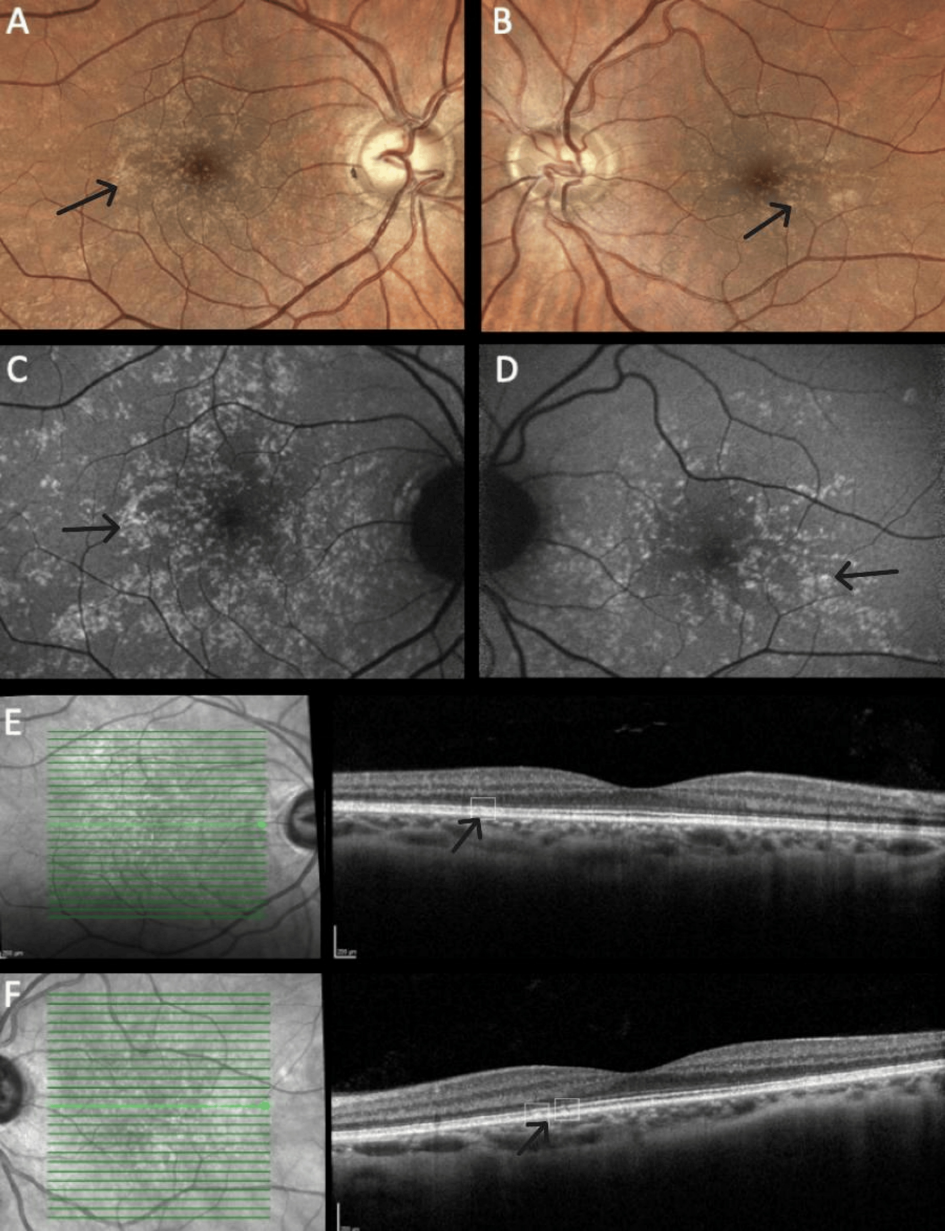
Multimodal imaging of patient #1. Color fundus photograph of the right (A) and left (B) eyes. Note the presence of small yellow dots in both maculas with similar patterns and distributions. Fundus autofluorescence of the right (C) and left eyes (D) showing the dots' characteristic hyperautofluorescence. Optical coherence tomography is globally normal with minor outer retinal irregularities, respectively, on the right (E) and left (F) eyes (annotated).

**Figure 2:**
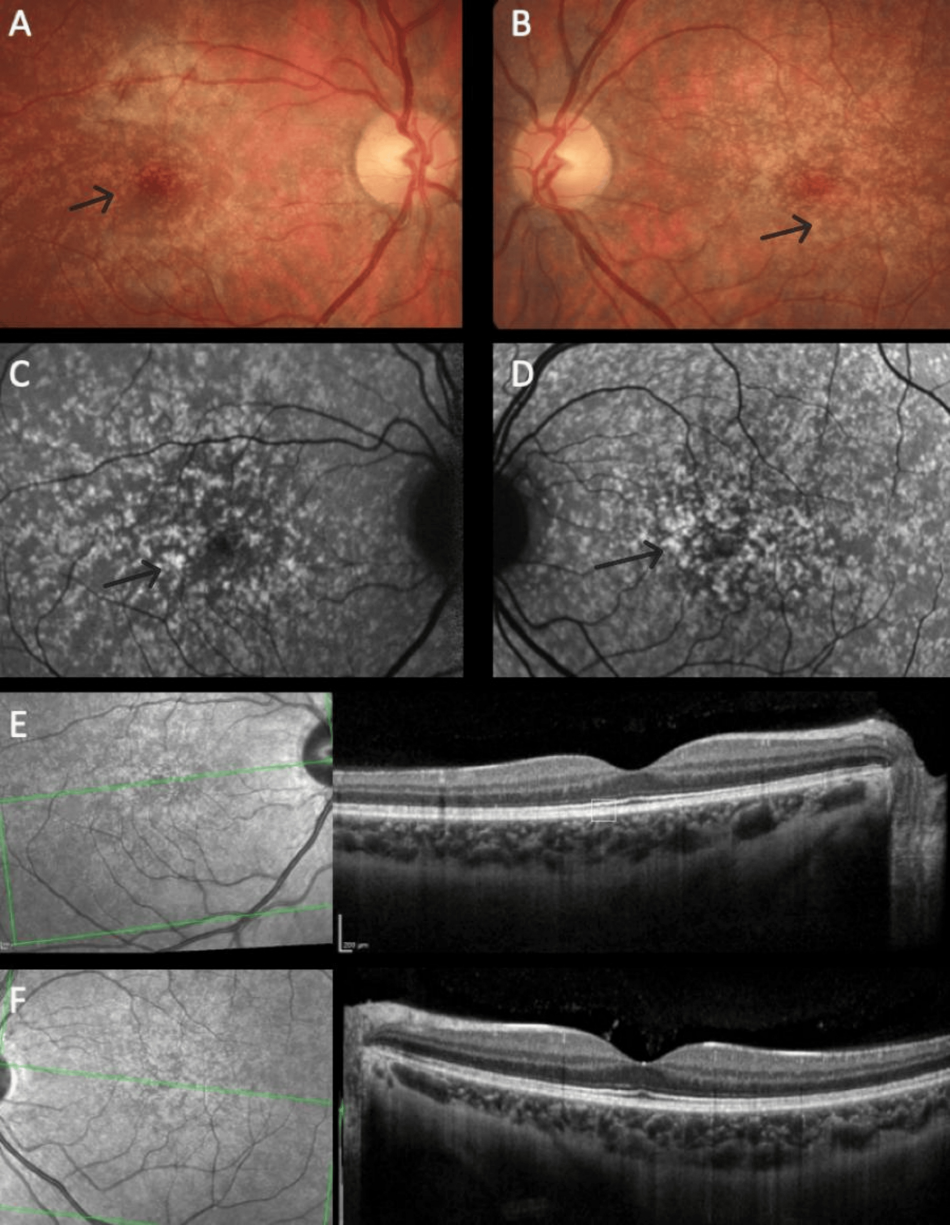
Multimodal imaging of patient #2. Color fundus photograph of the right (A) and left (B) eyes. In both maculas, multiple small dots are evenly distributed. Fundus autofluorescence of the right (C) and left eyes (D) demonstrating the dots' hyperautofluorescence. Optical coherence tomography of the right (E) and left (F) eyes is globally unremarkable with minor irregularities (annotated).

**Figure 3:**
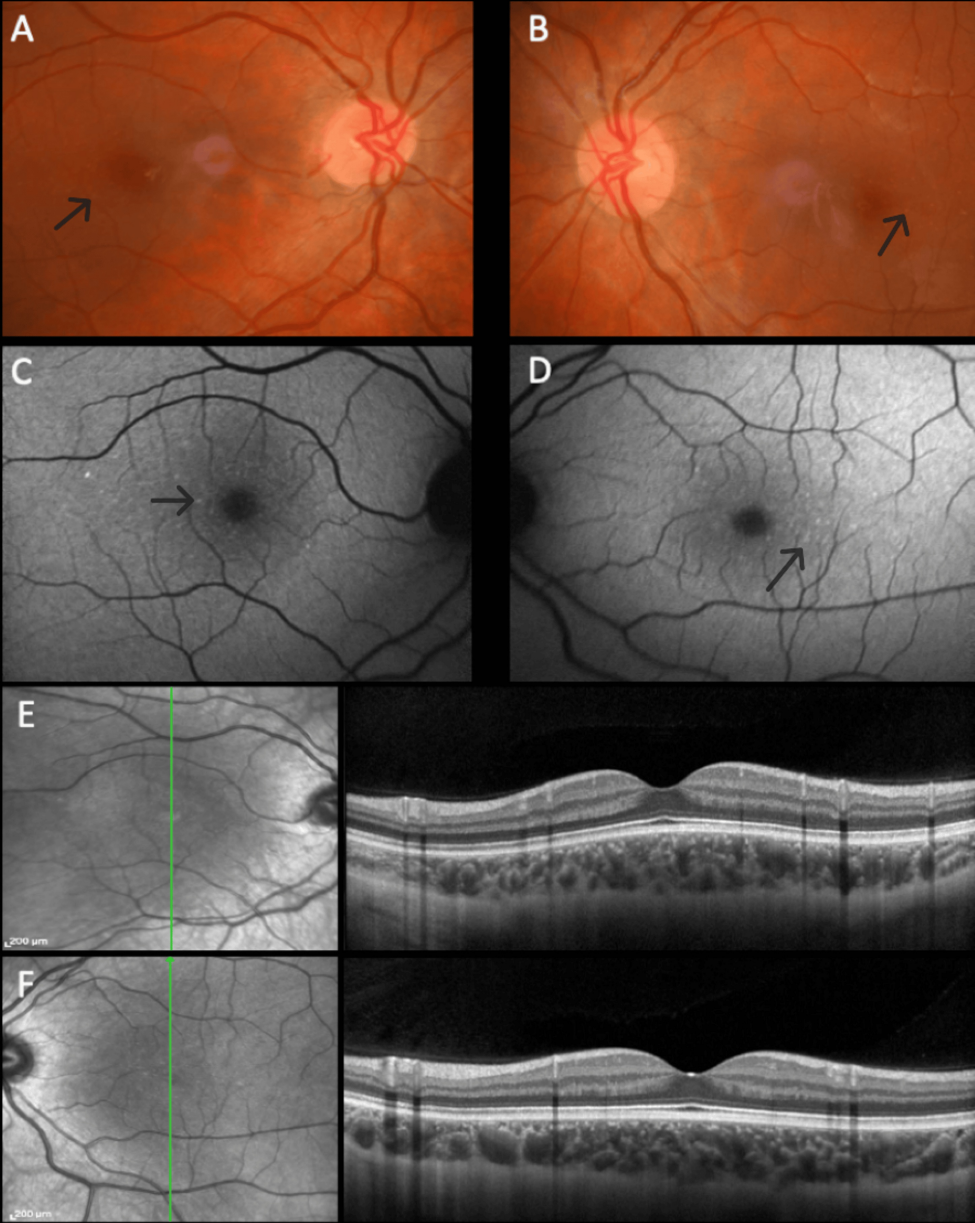
Multimodal imaging of patient #3. Color fundus photograph of the right (A) and left (B) eyes. Dots are small and few, distributed around the fovea. Fundus autofluorescence of the right (C) and left (D) eyes reveals hyperautofluorescent dots. Optical coherence tomography was globally normal in the right (E) and left (F) eyes.

**Figure 4:**
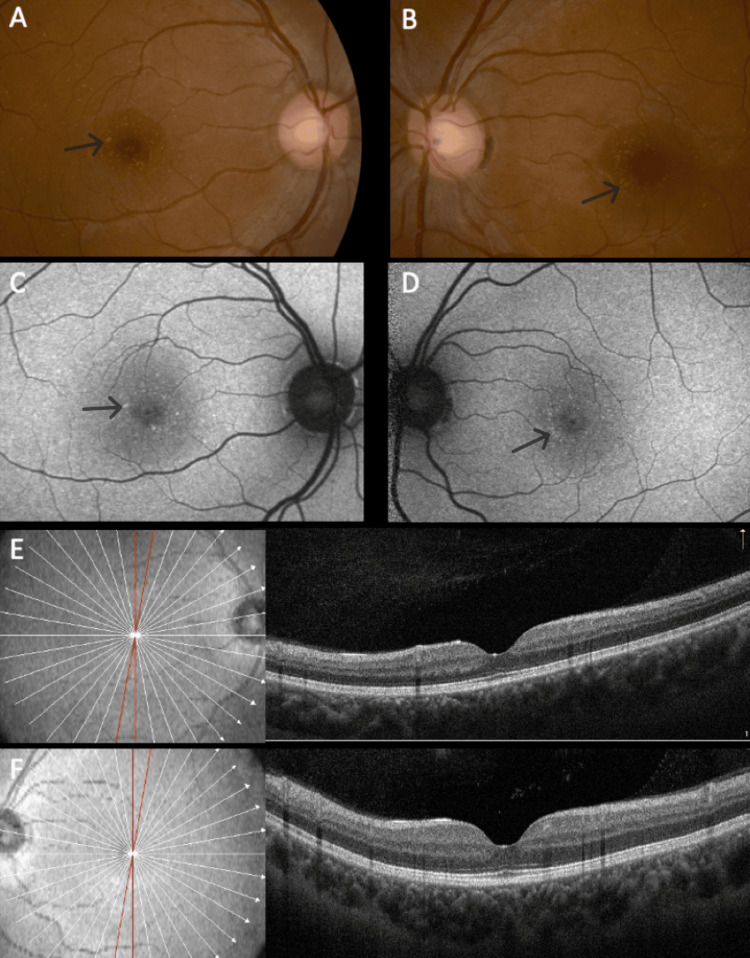
Multimodal imaging of patient #4. Color fundus photograph of the right (A) and left (B) eyes. Note the presence of small yellow dots in both maculas. Fundus autofluorescence of the right (C) and left eyes (D) showing the dots' typical hyperautofluorescence. The dots are mostly inapparent in optical coherence tomography, respectively, right (E) and left (F) eyes.

**Figure 5:**
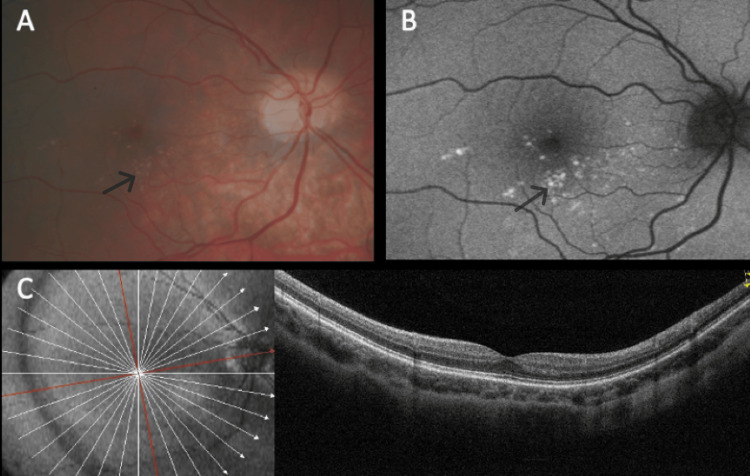
Multimodal imaging of the right eye of patient #5. Color fundus photograph (A). Note the presence of the dots in the nasal and inferior macula. The fundus autofluorescence (B) shows matched hyperautofluorescence of the lesions. The optical coherence tomography (C) is unremarkable.

The distribution pattern of yellow dots varied among patients. In patients #1 and #2, the pattern was similar, with a uniform distribution around the fovea (Figures 1A, 1B), even though in patient #2, overlapping areas of hypopigmentation could be identified (Figures 2A, 2B). The same pattern was observed on FAF imaging with the dots being hyperautofluorescent in both subjects (Figures 1C, 1D, 2C, 2D). In patient #3, the dots were conglomerated in the nasal parafovea of the right eye and in both eyes were in the temporal parafovea and extended to the perifovea (Figures 3A, 3B) and FAF revealed scarce hyperautofluorescent dots (Figures 3C, 3D). In patient #4's right eye, the dots were equally distributed in the parafovea with some conglomerates (Figure 4A), while in the left eye, they concentrated more in the nasal region of the parafovea (Figure 4B). FAF revealed multiple areas of hyperautofluorescence congruent with fundoscopic findings (Figures 4C, 4D). In patient #5, most dots were concentrated in the inferior nasal region of the parafovea, with some confluent dots in the temporal region (Figure 5A) and FAF showed the dots more clearly (Figure 5B).

In all patients, OCT was globally normal albeit with some irregularities in the EZ and RPE (Figures 1E, 1F, 2E, 2F, 4E, 4F, 5C).

**Figure 6:**
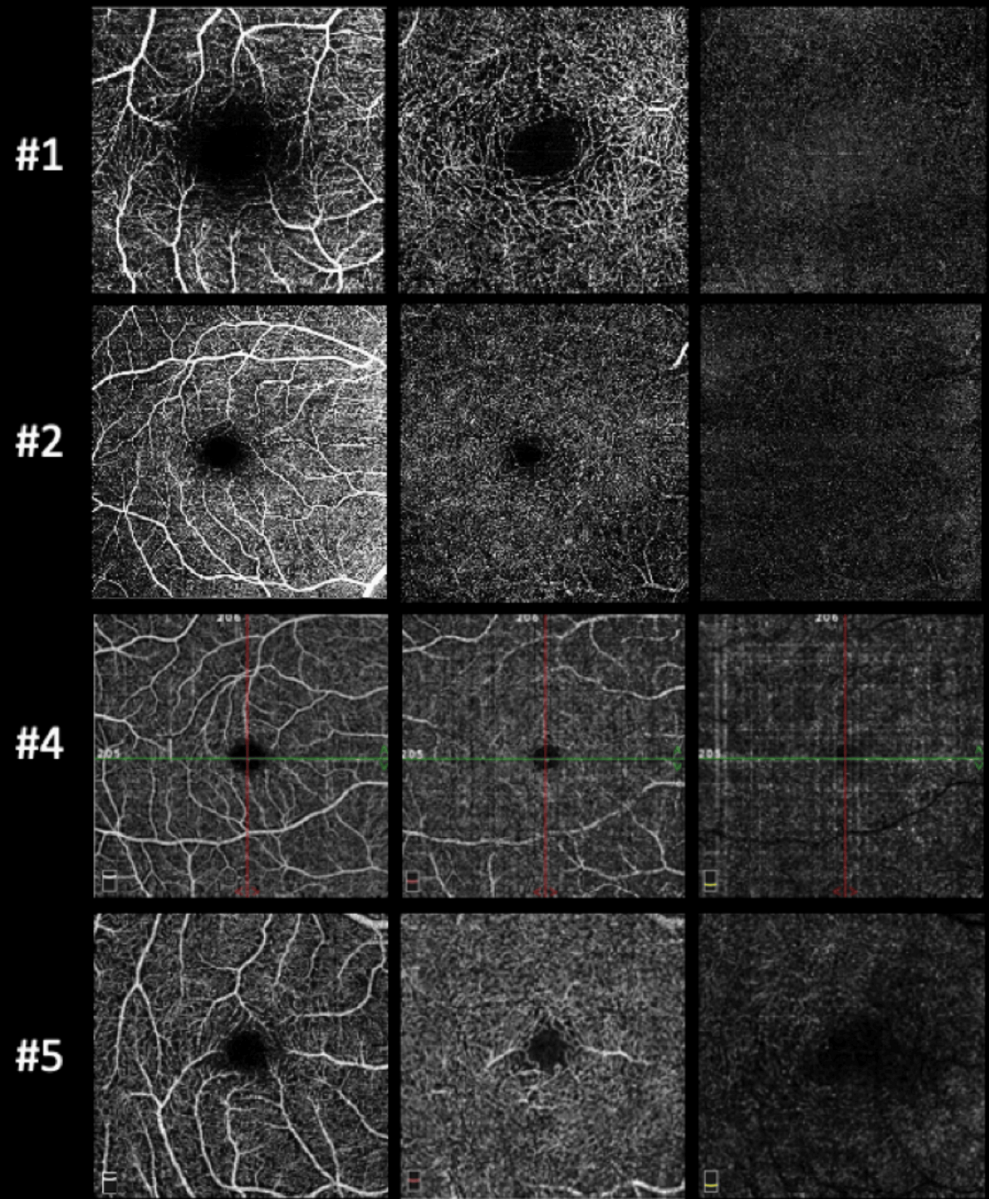
Optical coherence tomography angiography of the right eye of patients #1, #2, #4, and #5. From left to right, it is possible to observe the en-face image of the superficial, deep, and outer retina plexus, which were normal. The same for the left eye of patients #1, #2, and #4 (not shown).

OCT-A was normal in all patients who underwent the exam (Figure 6). The retinal periphery and vasculature were normal in all subjects. All the findings regarding the yellow dots remained stable during follow-up, demonstrating no progression of the lesions.

## Discussion

Dev Borman et al. published in 2017 the first description of 36 cases of BYDM as a novel childhood-onset macular phenotype that is non-progressive and does not affect macular function (the largest cohort to date). Since then, five further case reports have been published [2-6], including the first unilateral case reported by Mishra et al. in 2021 [2].

In this report, we presented a series of five cases of BYDM from three Portuguese centers. The ocular history of our cohort was diverse and unrelated to the presence of yellow dots. On examination, characteristic macular changes were observed, consisting of multiple small yellow dots at the level of the RPE around the fovea. The dots were evenly distributed throughout the macula or preferentially conglomerated in the nasal region. This distribution pattern of the dots was consistent with what is known in the literature [4]. Four out of five patients showed bilateral dots, with the exception being patient #5, the second case of unilateral BYDM described in the literature thus far [2].

As previously reported, these dots were hyperautofluorescent on FAF imaging in all cases, which appears to be a hallmark of this macular phenotype. The dots were small and did not show progression on clinical evaluation of fundus photographs, which makes it unlikely that there is a significant visual impact. As expected, OCT and OCTA were globally normal although some small irregularities could be noted in the EZ and RPE. No abnormalities were found at the level of inner retinal layers, as seen in the white dot fovea phenotype, which is similar but characterized by hyperreflective granulation in the inner retinal layers [4,7].

Within our patient cohort, we observed significant demographic variability. The mean age of our patients was 31 years, which is relatively higher than the average age of 16 years reported in all 46 known cases to date [5]. We speculate that this age difference may be attributed to two patients who went undiagnosed due to a lack of knowledge about this phenotype. All our patients were female, in contrast to previous reports where male cases were also present. However, given the relatively small sample size, it is difficult to draw a conclusion regarding the correlation between gender and disease prevalence. None of our patients were symptomatic and VA remained unchanged between the first and last examination as was expected.

Limitations of our study include its retrospective nature and the small number of cases, although we present the third-highest number of cases in the literature. There is also a selection bias, as only selected cases from three Portuguese centers are reported, without a comprehensive survey of all possible diagnosed or underdiagnosed cases.

## Conclusions

We conclude that BYDM is a rare phenotype characterized by the presence of bilateral or unilateral small white-yellow dots encircling the fovea, as identified through routine fundus examination or familial screening. Their stable clinical status during long-term follow-up confirms the benign and stationary nature of this phenotype. Multimodal imaging techniques, such as OCT, OCTA, and FAF, are helpful in its diagnosis. This study expanded on the small body of knowledge on BYDM and characterized the first five Portuguese cases, including the second described unilateral case globally.

## References

[1] Dev Borman A, Rachitskaya A, Suzani M (2017). Benign yellow dot maculopathy: a new macular phenotype. Ophthalmology.

[2] Mishra AV, Pollmann AS, Choudhry N, Demmings E, Gupta RR (2021). Unilateral benign yellow dot maculopathy. Am J Ophthalmol Case Rep.

[3] Moisseiev E (2018). Benign yellow dot maculopathy. Am J Ophthalmol Case Rep.

[4] Murro V, Mucciolo DP, Giorgio D (2019). Multimodal imaging of benign yellow dot maculopathy. Ophthalmic Genet.

[5] Kasetty VM, Desai TU, Desai UR (2023). Benign yellow-dot maculopathy: case report and review of the literature. Can J Ophthalmol.

[6] Ninet L, David T, Gascon P (2022). Multimodal imaging for benign yellow dot maculopathy. Ophthalmol Retina.

[7] Witkin AJ, London NJ, Wender JD, Fu A, Garg SJ, Regillo CD (2012). Spectral-domain optical coherence tomography of white dot fovea. Arch Ophthalmol.

